# A Newton-based shooting method to find synaptic threshold in active cables

**DOI:** 10.1186/1471-2202-16-S1-P116

**Published:** 2015-12-18

**Authors:** William L Kath

**Affiliations:** 1 Departments of Applied Mathematics and Neurobiology, Northwestern University, Evanston, IL 60201, USA

## 

The integration of synaptic inputs in a neuron can be nonlinear not just at the axon, but also locally in the dendrites if they are imbued with active voltage-gated ion channels. For example, CA1 pyramidal neurons have high densities of sodium and potassium and currents in their dendrites, and these densities can vary substantially in the arbor [1,2]. Such nonlinearities can lead to compartmentalized responses to inputs [3], with branches acting as individual nonlinear units in which dendritic spikes occur. A cell can thus function as a multi-layered network with the soma as final output. This motivates determining when a given set of synaptic inputs is large enough to generate a local dendritic spike, or, alternatively, determining the synaptic conductance value(s) at threshold for producing a spike.

Above- and below-threshold conditions are known to be separated by the threshold or critical surface [4]. Here it is shown that the synaptic conductance leading to a threshold solution can be found by modifying Newton methods developed to find steady-state solutions in fluid mechanics [5]. Consider a general form of the cable equation,

$$\frac{\partial u}{\partial t}=L\left(x\right)u+N\left(u,x\right)+g_{s}G_{s}\left(t,x\right)\left(u_{\text{rev}}-u\right)$$

Here **u **represents the voltage and any gating variables. The first term on the right models the diffusive part of the cable equation, and the next term the nonlinearities from any active voltage-gated ion channels. The last term represents synaptic conductances at points in the dendritic tree with overall strength *g_s_*. An unstable threshold solution and accompanying synaptic strength can be found using 1) a preconditioned version of the steady-state cable equation combined with constraints requiring the difference between a shooting solution from rest, **u**(*T*,**x**;*g_s_*), and the critical surface to be perpendicular to the single unstable eigenvector associated with the critical surface. The overall procedure finds the value of *g_s _*leading to a solution that asymptotes to the critical surface as *t *goes to ∞.

**Figure 1:**
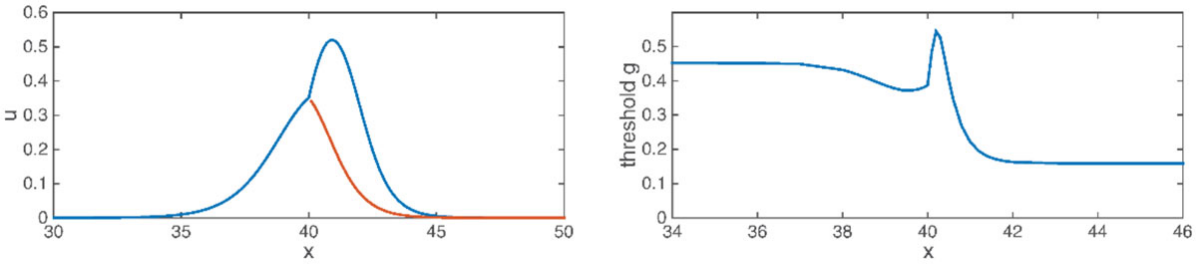
**Threshold Fitzhugh-Nagumo voltage profile in a branched cable for synapse position just past the branch point (left; blue is main and one daughter branch; red is the other) and critical conductance value as a function of synapse position in main and one daughter branch (right)**. The branch point is at position × = 40.

Example results from the method are shown in Figure 1. These are for the Fitzhugh-Nagumo model without recovery in a y-branched cable morphology where the daughter branches have half the diameter of the main dendrite. One observes the expected drop of threshold conductance as the synapse moves from the main to daughter branch, but this transition is not monotonic as for a passive cable because of the nonlinear active conductance in the model. Discovering phenomena such as this would be quite laborious without employing a Newton-based method.
